# Cathodal HD-tDCS on the right V5 improves motion perception in humans

**DOI:** 10.3389/fnbeh.2015.00257

**Published:** 2015-09-23

**Authors:** Giuseppe A. Zito, Theresa Senti, Dario Cazzoli, René M. Müri, Urs P. Mosimann, Thomas Nyffeler, Tobias Nef

**Affiliations:** ^1^Gerontechnology and Rehabilitation Group, University of BernBern, Switzerland; ^2^Division of Cognitive and Restorative Neurology, Department of Neurology, University Hospital Inselspital, University of BernBern, Switzerland; ^3^University Hospital of Old Age Psychiatry and Psychotherapy, University of BernBern, Switzerland; ^4^Center of Neurology and Neurorehabilitation, Luzerner KantonsspitalLuzern, Switzerland; ^5^ARTORG Center for Biomedical Engineering Research, University of BernBern, Switzerland

**Keywords:** HD-tDCS, motion perception, shape perception, unilateral brain stimulation, visual test

## Abstract

Brain lesions in the visual associative cortex are known to impair visual perception, i.e., the capacity to correctly perceive different aspects of the visual world, such as motion, color, or shapes. Visual perception can be influenced by non-invasive brain stimulation such as transcranial direct current stimulation (tDCS). In a recently developed technique called high definition (HD) tDCS, small HD-electrodes are used instead of the sponge electrodes in the conventional approach. This is believed to achieve high focality and precision over the target area. In this paper we tested the effects of cathodal and anodal HD-tDCS over the right V5 on motion and shape perception in a single blind, within-subject, sham controlled, cross-over trial. The purpose of the study was to prove the high focality of the stimulation only over the target area. Twenty one healthy volunteers received 20 min of 2 mA cathodal, anodal and sham stimulation over the right V5 and their performance on a visual test was recorded. The results showed significant improvement in motion perception in the left hemifield after cathodal HD-tDCS, but not in shape perception. Sham and anodal HD-tDCS did not affect performance. The specific effect of influencing performance of visual tasks by modulating the excitability of the neurons in the visual cortex might be explained by the complexity of perceptual information needed for the tasks. This provokes a “noisy” activation state of the encoding neuronal patterns. We speculate that in this case cathodal HD-tDCS may focus the correct perception by decreasing global excitation and thus diminishing the “noise” below threshold.

## Introduction

The visual cortex is the region of the brain responsible for visual perception. It is divided into the primary visual cortex V1, anatomically equivalent to Brodmann Area (BA) 17, and the extrastriate visual cortical areas V2, V3, V4, and V5, corresponding to BA 18 and 19 (Engel et al., 1997; Van den Stock et al., 2014). These visual areas are organized into two hierarchically and functionally specialized processing pathways: a ventral “what” stream, including V1, V2, V4 and the inferior temporal areas TEO and TE, for object vision; and a dorsal “where” stream, including V1, V2, V3, the middle temporal area (V5-MT), the medial superior temporal area (MST), and further stations in the inferior parietal and superior temporal sulcal cortex, for spatial vision and motion perception (Ungerleider and Haxby, 1994; Huberle et al., 2012).

Visual perception can be influenced by non-invasive brain stimulation. Transcranial direct current stimulation (tDCS), for instance, is a technique widely used to influence the neuronal excitability (Antal et al., 2001). tDCS is performed by applying a constant low current, delivered to the brain area of interest via external electrodes. Typical stimulation parameters are 1–2 mA for a duration of up to 20 min (Nitsche et al., 2008). High Definition tDCS (HD-tDCS) is a recently developed method (Villamar et al., 2013), in which small HD-electrodes are used instead of the two large sponge electrodes in the conventional approach. Compared to the conventional approach, HD-tDCS has the advantage to give much higher focality over the target region (Bikson et al., 2012). A typical montage for HD-tDCS is the 4 × 1 ring configuration, in which a central electrode is placed over the target region, and four return electrodes are placed around it in a ring-shape configuration (Datta et al., 2009). Many studies have investigated the effects of tDCS on the visual cortex. For instance, Antal et al. reported reduced phosphene thresholds after 10 min of 1 mA anodal tDCS and increased phosphene threshold after cathodal tDCS over Oz in the occipital pole (Antal et al., 2003). Using a similar protocol, Accornero et al. studied the tDCS-induced modifications in visual evoked potentials (VEP-P100) in humans, and found that anodal polarization reduced VEP-P100 amplitude whereas cathodal polarization significantly increased amplitude (Accornero et al., 2007). This suggests that, according to the polarity of the stimulation, anodal and cathodal tDCS elicits different effects. Anodal tDCS is known to cause a depolarization of the resting membrane potential in the neurons, which increases excitability; whereas cathodal tDCS causes a hyperpolarization of the resting membrane potential, with a decrease of the neuronal excitability (Nitsche et al., 2008). However, some studies have found contradictory behavioral effects and different theories have been proposed to justify such results (Antal et al., 2004; Batsikadze et al., 2013). Antal et al., for instance, showed that conventional cathodal tDCS over the left V5 affected a visuomotor task by modifying only visual perception, and controlled it by stimulating different areas of the visual and motor cortex; anodal tDCS did not affect behavior. The effects depended also on task difficulty. But, to our knowledge, no studies with HD-tDCS over the right V5 have been conducted up to date. Given the task- and location-dependent effects of tDCS (Nitsche et al., 2008), the theory about the depolarization and hyperpolarization of the neurons is somewhat too ingenuous to explain the complexity of the problem. Behavioral performance on a visual motion task after stimulation of the right V5 needs to be further explored.

In the present paper, we investigated the influence of HD-tDCS on motion and shape perception. HD-tDCS was applied over the right V5 of healthy volunteers, and the performance on visual tests was recorded. The aim of the study was to investigate the focality of HD-tDCS using known properties of distinct areas of the visual cortex (i.e., the visual motion processing area located in V5), and to examine polarity-dependent inhibitory and excitatory effects of HD-tDCS on behavioral performance. Two hypotheses were tested: first, if HD-tDCS focuses the stimulation on V5 only, a behavioral effect in only motion perception should be observed. Other properties of objects, such as shape, are processed in distinct, although near V5, areas, and should therefore not be influenced by HD-tDCS. Second, anodal and cathodal HD-tDCS on the visual cortex do not provoke excitatory and inhibitory effects, respectively, on behavioral performance.

## Material and Methods

### Participants and Ethical Approval

Twenty one healthy volunteers (11 men and 10 women, 12 right handed, mean age = 30.5, SD = 5.1 years) were recruited to participate in the study. All subjects had at least a Bachelor Diploma and were experienced computer users. The inclusion criterion was a visual acuity of > 0.8, corrected with lenses if needed. Exclusion criteria were: serious head injuries, seizures, frequent or severe headaches, metal pieces in the body, and implanted medical devices (Villamar et al., 2013). None of the subjects was taking any medication at the time of the study.

The study was carried out in accordance with the latest version of the Declaration of Helsinki, and ethical approval was provided by the Ethics Committee of the Canton of Bern, Switzerland.

### Experimental Design

**Figure 1:**
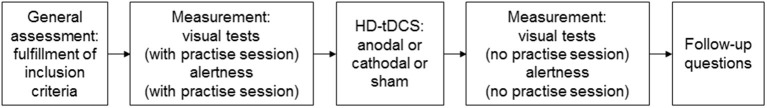
**Experimental design of the single blind within-subject, sham controlled randomized cross-over trial**.

The study was designed as a single blind, within-subject, sham controlled, randomized, cross-over trial. Prior to the study, all subjects gave written informed consent. Subjects performed a practice session of the visual tests, followed by the actual testing session. As a control task, subjects also performed an alertness task, preceded by a corresponding practice session (Zimmermann and Fimm, 2002). The stimulation (anodal, cathodal, or sham) was then administered. Right after the stimulation, the subjects then repeated the visual tests and the alertness task, this time without practice sessions. Performance during the stimulation was not measured. In this way, participants could make a break between the two sessions and an attention decrease due to tiredness was avoided. Finally, they were asked about potential adverse effects, such as headache, nausea, pain, or trouble concentrating (Villamar et al., 2013). Moreover, they were asked to state whether they believed to have received sham or real stimulation. The experimental design is depicted in Figure 1.

The duration of the experiment was about 1 h, roughly divided in the following way: 5 min for general assessment, 15 min for first measurement, 5 min to prepare the subjects for the stimulation, 20 min stimulation, and 15 min for the second measurement. The second and the third sessions took place at least 1 week after the previous session, respectively, and at the same time of the day. The order of the stimulation conditions in the respective sessions was counterbalanced across subjects.

### Test Setup

**Figure 2:**
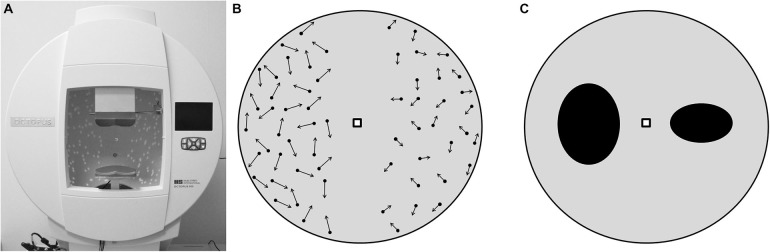
**Visual tests setup, as it appeared to the subjects. (A)** hemispherical screen used to perform the tests. **(B)** Speed Task. The arrows represent the speed of the dots, which, in this example, are faster in the left hemisphere. Here subjects are asked to decrease the speed of the dots on the left in order to make them as fast as the dots on the right. **(C)** Shape Task with two ellipses, the axes ratio of the ellipse in the left hemisphere is higher than the one in the right. Here subjects are asked to decrease the axes ratio of the ellipse on the left in order to make it identical to the ellipse on the right.

For the visual tests, subjects were seated with their head resting on a chin- and forehead rest at the center of a hemispherical screen (cupola, Figure 2A), where visual stimuli were projected, and held an input device with three buttons in their dominant hand; two buttons to manipulate the images and one to confirm the choice. The tests followed a balance paradigm, in which subjects had to compare two images presented on the left and the right half of the cupola, respectively, at an eccentricity of 7.6° visual angles (VA) from its center, while fixating a central marker point (0° VA eccentricity). In this setup, the two halves of the hemispherical screen corresponded to the two visual hemifields (VH). Two different subtasks, with 12 repetitions per task, were administered in a pseudo-random order. In the first one, called the Speed Task, two patterns of dots moving in random direction at two different speeds were presented. The subjects were asked to consider the speed of the dots on the right VH as reference, and to use the two buttons of the input device to change (increase or decrease) the speed of the dots on the left VH until they matched the reference (Figure 2B). Reference speeds were 0.36° VA/s, 1.07° VA/s, 1.80° VA/s, and 2.52° VA/s. In the second subtask, called the Shape Task, two ellipses with different ratios between the vertical and the horizontal axes, were presented. The subjects were asked to consider the ellipse on the right VH as reference, and to change the ratio of the ellipse on the left VH until it was perceived as identical as the one on the right VH (Figure 2C). Reference ratios were 0.3, 0.6, 1.5 and 1.8.

The visual tests were performed under central fixation, which was controlled using an eye tracking system integrated in the hemispherical screen. If the subjects moved their gaze outside an allowed region of ±5° VA from the central fixation marker, the visual stimuli disappeared, and they only reappeared when the central marker was fixated again. The technical setup is described in detail elsewhere (Zito et al., 2014).

Alertness was measured with the Test of Attentional Performance (TAP; Zimmermann and Fimm, 2002). In this test, a cross appeared on a computer flat screen at randomly varying time intervals, and the subjects were asked to respond to it as quickly as possible by pressing a key.

Both the visual tests and the TAP were run on an Intel^®^ Core^TM^ i5 (3.10 GHz) with Windows 7 operating system (Microsoft Inc.). The monitor of the computer for the TAP test was a 24′ screen with a resolution of 1920 × 1080 pixels.

### HD-tDCS Stimulation

**Figure 3:**
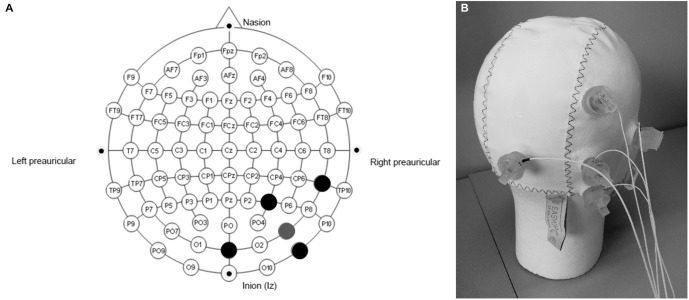
**Electrodes montage for the High Definition transcranial direct current stimulation (HD-tDCS) experiment. (A)** the gray point represents the central electrode, placed over PO8; the black points represent the other four (return) electrodes, placed over P4, OZ, TP8 and PO10. **(B)** Easycap, with electrodes placed according to the selected montage.

The selected target region for the stimulation was V5. HD-tDCS was administered using a battery-driven, constant-current generator (DC-Stimulator MC, neuroConn GmbH, Germany), connected to a passive HD-tDCS distributor (Soterix Medical, NY, USA). The optimal electrodes montage was selected by means of a bioelectromagnetic simulator of the current flow into the brain (Soterix HD-Explore, Soterix Medical, NY, USA). This software uses a finite element method to compute the distribution of the electrical field into a standard adult male head model, once the location of the electrodes and the stimulation parameters are given. Optimization criteria for the software were high focality and high field intensity in the target area. According to the solution of the optimization problem, the following montage was selected: the HD-tDCS cathode casing (Minhas et al., 2010) was placed in PO_8_ and four anode casings were placed at a distance of about 5 cm from the cathode, their location corresponding to P_4_, OZ, TP_8_, and PO_10_ according to the 10–10 standard EEG system (Figure 3). With this montage, the central electrode is right above V5 (Dumoulin et al., 2000). In order to increase conductivity, the hair under the casings was separated to expose the scalp skin, and about 3 ml of Signa Gel (Parker Laboratories, NJ, USA) were injected into the electrode casings. The electrodes were then placed into the gel solution inside the casings, and held in place with the casing cap (Easycap GmbH, Germany) (Borckardt et al., 2012). Impedance values were examined for each of the 5 electrodes and were all verified to be < 6 kΩ for the duration of the entire session. For real HD-tDCS, the current was ramped up to 2 mA (ramp duration of 30 s and maintained for 20 min. For sham HD-tDCS, the current was ramped up to 2 mA (ramp duration of 30 s and, after 1 min of full stimulation, it was ramped down to 0 mA (ramp duration of 30 s, and stayed off until the end of the session. This helped to mask sham and real conditions and gave to the participants a few seconds to adapt to the tickling sensation of the current.

### Data Analysis

The distribution of the electrical field into the brain was graphically analysed with the bioelectromagnetic simulator (Soterix HD-Explore, Soterix Medical, NY, USA).

For the visual tests, the performance per subject was the mean value, out of the 12 repetitions, of the ratio between the two images at the very time when the confirmation button was pressed. In particular, for the Shape Task it was the ratio between the eccentricity of the ellipse under manipulation and the eccentricity of the reference ellipse. For the Speed Task it was the ratio between the speed of the dots under manipulation and the speed of the reference dots. The performance on the TAP—Alertness Task was expressed in terms of mean reaction time following target stimulus presentation. Tukey Test with scaling factor 1.5 was used to identify potential outliers (Richmond, 2000).

The influence of HD-tDCS on the performance was assessed with Repeated Measures Analysis of Variance (rmANOVA) with time (pre, post) × condition (sham, cathodal, anodal) × task (Speed Task, Shape Task) as within-subjects factors, gender (male, female) and handedness (right, left) as between-subjects factors. Alertness was tested with a rmANOVA with time (pre, post) × condition (sham, cathodal, anodal) as within-subjects factors, gender (male, female) and handedness (right, left) as between-subjects factors. Tukey’s HSD tests were used for *post hoc* comparisons.

Chi-square tests were used to evaluate the number of subjects correctly guessing whether they received real or sham HD-tDCS (Borckardt et al., 2012). Data were analyzed using STATISTICA 8.0 (StatSoft Inc.).

## Results

### HD-tDCS Stimulation

**Figure 4:**
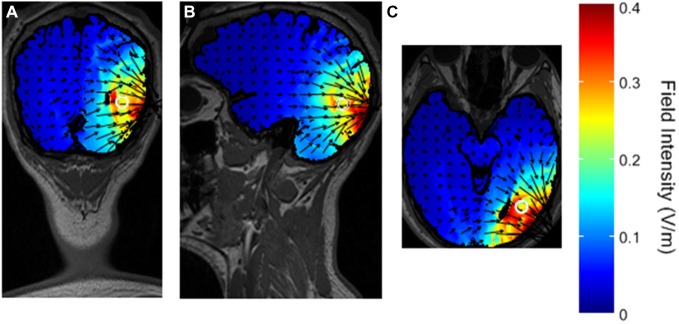
**Simulated distribution of the electrical field in the brain with the selected montage**. The white circle represents the presumed V5 in the head model. **(A)** coronal view. **(B)** sagittal view. **(C)** axial view.

The simulated distribution of the electrical field in the brain with the selected montage is shown in Figure 4.

The maximal intensity of 0.4 V/m is located in the right visual cortex, on the Brodmann Area (BA) 18 and 19. The surrounding BA 17, 37, 39, and 7 are only partially stimulated, and the maximal field intensity in those regions is about 0.2 V/m, reaching a depth of a few centimeters. These results are in line with previous studies using HD-tDCS in the 4 × 1 ring electrodes configuration (Borckardt et al., 2012; Villamar et al., 2013).

### Performance on the Visual Tests

All subjects tolerated well HD-tDCS, without any side effects (such as headache, pain, nausea, or trouble concentrating). For both sham and real conditions, the subjects frequently reported perceiving a tickling sensation, but only at the very beginning of the stimulation session, and then they rapidly adapted to this sensation. None of the subjects was aware of the expected effects of HD-tDCS. The results of the Chi-square tests with respect to the blinding of the study showed that subjects were not able to correctly guess above chance whether they received real or sham stimulation (*X*^2^[1] = 2.85, *p* > 0.05 for the first session, *X*^2^[1] = 2.74, *p* > 0.05 for the second session; *X*^2^[1] = 2.76, *p* > 0.05 for the third session).

**Figure 5:**
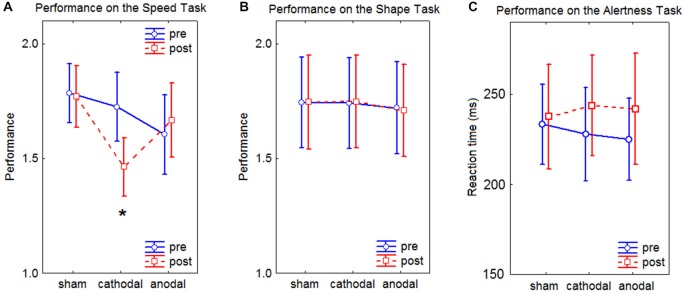
**Line charts of the results of the Speed Task, the Shape Task, and the Alertness Task**. In the graphs, the performance represents the ratio between the two compared images. Values close to 1 depict high performance. Error bars represent the standard error of the mean. **(A)** results of the Speed Task, the asterisks depicts a significant difference at *p* < 0.05 as assessed by Tukey’s HSD *post hoc* tests. **(B)** results of the Shape Task. **(C)** results of the Alertness Task.

The results of the visual tests and the alertness test are shown in Figure 5.

RmANOVA on the performance revealed a significant effect of interaction time × condition × task (*F*_(2,19)_ = 7.76, *p* = 0.002). *Post hoc* tests revealed a significant improvement in the performance for the Speed Task after cathodal HD-tDCS (*p* < 0.05). No significant effects of interaction were found in the performance on the Alertness Task (*F*_(2,19)_ = 1.03, *p* = 0.37). No main effects of Gender and Handedness were observed in the performance on the visual tests (*F*_(1,20)_ = 0.38, *p* = 0.54) for Gender, (*F*_(1,20)_ = 0.01, *p* = 0.92) for Handedness, nor in the Alertness (*F*_(1,20)_ = 0.89, *p* = 0.36) for Gender, (*F*_(1,20)_ = 1.92, *p* = 0.18) for Handedness.

## Discussion

The aims of the present paper were to investigate focality of HD-tDCS and to find evidence of the effects of tDCS on behavioral performance. The tested hypotheses were: first, HD-tDCS over the right V5 affects motion perception, but not shape perception; second, cathodal and anodal HD-tDCS do not induce decreased and increased performance, respectively, on behavioral tasks.

### Performance on the Visual Tests

The results of the visual tests showed a significant effect of cathodal HD-tDCS on the motion perception task. Performance during stimulation was not measured. However, in other studies on the visual cortex with during- and immediately after-stimulation protocols, the results did not show differences between the two time points measures (for a review, see Antal et al., 2006) and the conclusions were not affected by this additional information. According to the organization of visual processing into a “what” and a “where” stream (Ungerleider and Haxby, 1994), a visual test involving motion perception, like the Speed Task, would critically rely on areas of the “where” stream, such as V5. Conversely, a test involving perception of shapes, like the Shape Task, would critically rely on areas of the “what” stream. Our montage was selected to specifically target area V5, and this localization was supported by the simulated distribution of the electrical field (Figure 4).

While the individual distribution of the field in the brain of single subjects is not known, the pattern of our results supports the focality of stimulation over V5 only, as behavioral performance was affected only in the motion perception task (Speed Task). Stimulation in other areas, namely the “what” stream, may thus not be strong enough to elicit behavioral effects in the shape perception task (Shape Task). Furthermore, it has been shown in studies on the motor cortex that a maximum field intensity of 0.4 V/m elicits behavioral changes (Borckardt et al., 2012). No studies with lower field intensities have been reported, suggesting that such intensities have no effects. According to Figure 4, brain areas around V5 also involved in motion processing, like the posterior parietal cortex (Koch et al., 2008) or V1, were stimulated with a field intensity < 0.2 V/m, and this may not be sufficient to provoke behavioral changes.

The results of the Alertness Task showed no difference between stimulation conditions. This indicates that the improvement of behavioral performance in the Speed Task is not due to unspecific stimulation effects, such as changes in the alertness level.

This effect of enhancing behavioral performance by inhibiting cortical activity was highly specific and, apparently, paradoxical. However, such effects have already been reported in a previous tDCS study: Batsikadze et al. found that cathodal tDCS might induce qualitatively different effects, depending on current intensities (Batsikadze et al., 2013); Antal et al. showed that, in a visuo-motor coordination task similar to our paradigm, cathodal tDCS over V5 ameliorated behavioral performance, whereas anodal stimulation had no effect (Antal et al., 2004).

**Figure 6:**
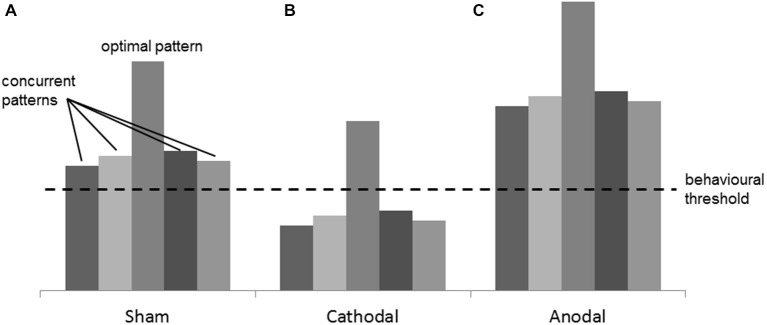
**Figure adapted from Antal et al. (2004)**. Example of the mechanism of interaction between tDCS and the cortical excitability. **(A)** in the Sham condition, many concurrent neuronal patterns, together with the optimal one, are simultaneously activated producing a “noisy” activation state. **(B)** in the Cathodal condition, the cortical excitability is globally decreased and the optimal pattern is the only one still above threshold, thus a focus effect is present. **(C)** in the Anodal condition, the global excitation of the concurrent patterns does not produce any change in the behavior because the “noise” is not filtered.

A speculative explanation of such effects was found in the complexity of perceptual information needed for the tasks. When high resolution, temporo-spatial analysis, and comparison of motion speeds and directions are involved, different encoding neuronal patterns in response to the different speeds and motion directions may activate simultaneously. This probably results in a globally “noisy” activation state, where optimal and suboptimal patterns are both present at the same time (Figure 6A). Hypothesizing a threshold in the neuronal activation, above which a behavioral change can be observed, Antal et al. speculated that cathodal stimulation may focus the correct perception of these parameters by decreasing global activation level. As a consequence of this, the amount of activation of concurrent patterns is diminished below threshold (Figure 6B; Antal et al., 2006). Similar argumentations can be made for the results of the present study. In our Speed Task, for instance, speeds of dots moving in several random directions are compared. The random directions of the dots might tune different groups of neurons, and this might result in the “noisy” activation state addressed by Antal et al. Here the optimal neuronal patterns represent the target speeds, and the suboptimal patterns represent the different motion directions of the dots. It is plausible that, after cathodal HD-tDCS, the neuronal activation state looks like the one shown in Figure 6B, where only the optimal pattern is still above threshold. Anodal stimulation would, on the other hand, increase the neuronal activation even more but, since the mentioned concurrent patterns are already above threshold, no effect on the performance is observed (Figure 6C). This explanation is supported by studies in macaque monkeys, where it has been demonstrated that different neurons in V5 show a high selectivity for different motion speeds and directions (Maunsell and Van Essen, 1983; Albright, 1984; Duijnhouwer et al., 2013).

### HD-tDCS Stimulation

When conducting HD-tDCS experiments, it is important to optimize the stimulation parameters, in order to achieve high focus and high field intensity over a given target region. The manipulated parameters to investigate this optimization problem are usually the shape and the size of the electrodes, and the inter-electrodes distance. It has been shown that ring-like electrodes decrease tickling sensation when compared with other shapes, such as pellets, rectangles, and disks (Minhas et al., 2010, 2011). The electrodes size is positively correlated with the current density in the brain (Miranda et al., 2009). Therefore, in order to achieve a strong field, bigger electrodes are desirable. Unfortunately, it is more difficult to reach high focality with large electrodes, as the electrical field propagates from a larger surface. For this reason, ring-shape HD-electrodes (Borckardt et al., 2012), which fulfill the above mentioned criteria, were selected.

The results of the Chi-square test supported indeed that the tickling sensation due to the stimulation did not affect the effectiveness of the blinding procedure, because subjects could not guess above chance whether they were receiving real or sham stimulation. It might be possible, however, that with slightly different protocols the difference in correct guessing would have been significant (Borckardt et al., 2012).

Regarding the distance among the electrodes, it has been shown that the higher the distance, the higher the field intensity (Faria et al., 2011), but, conversely, the lower the focus over the target region (Dmochowski et al., 2011). A good compromise was found with the montage proposed in the present study, in which the target electrode was placed over the visual area V5 (PO_8_ in the EEG standard 10–10 system), and the four return electrodes at the edge of the desired stimulation region (P_4_, OZ, TP_8_ and PO_10_).

### Strengths, Limitations and Outlook of the Present Study

The strengths of this study are the use of HD-tDCS instead of conventional tDCS to increase the focality of the stimulation, and the use of a bioelectromagnetic simulator to compute the distribution of the current into the brain, essential for HD-tDCS experiments. However, this method has limitations, because it only computes field distribution in a standard head, which could be different from the individual heads. In future studies, subject-specific head models derived from MRI, could be helpful.

Future research in the field should also conduct an in-depth study of the exact molecular mechanism of interaction between tDCS and the brain, because the question of how tDCS affects brain functioning, explored in recent publications (Stagg et al., 2013; Pirulli et al., 2014; Bikson, 2015), is still unanswered. In addition, other human factors which have been shown to play a role in visual perception, like anxiety and stress (Tyler and Tucker, 1982), or the presence of the menstrual cycle during the experiment (Ward et al., 1978), should be considered in the analysis, but this would require a much higher sample size, and was not the main goal of the current study. Task difficulty might also be manipulated, in order to see behavioral effects of tDCS only in demanding tasks (Antal et al., 2004).

## Author Contributions

GAZ carried out the technical implementation of the test and the stimulation setup. TS studied the feasibility of the test battery and collected the data from the subjects. DC helped in the statistical analysis of the results and in the discussion of the findings. RM helped in the conception of the test battery. UPM carried out the clinical evaluation of the experimental procedure. TN provided important feedbacks regarding the data analyses. TNef helped in the conceptual framework of the study design and coordinated the work. All authors contributed in designing the entire study, reading, correcting and approving the final manuscript.

## Conflict of Interest Statement

The authors declare that the research was conducted in the absence of any commercial or financial relationships that could be construed as a potential conflict of interest.

## Abbreviations

HD-tDCS: High Definition transcranial direct current stimulation
BA: Brodmann Area
MT: Middle temporal area
MST: Medial superior temporal area
VA: Visual angle
VH: Visual hemifield
TAP: Test of Attentional Performance.

## References

[B1] Accornero N. Li Voti P. La Riccia M. Gregori B. (2007). Visual evoked potentials modulation during direct current cortical polarization. Exp. Brain Res. 178, 261–266. 10.1007/s00221-006-0733-y 17051377

[B2] Albright T. D. (1984). Direction and orientation selectivity of neurons in visual area MT of the macaque. J. Neurophysiol. 52, 1106–1130. 6520628 10.1152/jn.1984.52.6.1106

[B3] Antal A. Kincses T. Z. Nitsche M. A. Paulus W. (2003). Manipulation of phosphene thresholds by transcranial direct current stimulation in man. Exp. Brain Res. 150, 375–378. 10.1007/s00221-003-1459-8 12698316

[B4] Antal A. Nitsche M. A. Kruse W. Kincses T. Z. Hoffmann K.-P. Paulus W. (2004). Direct current stimulation over V5 enhances visuomotor coordination by improving motion perception in humans. J. Cogn. Neurosci. 16, 521–527. 10.1162/089892904323057263 15165345

[B5] Antal A. Nitsche M. A. Paulus W. (2001). External modulation of visual perception in humans. Neuroreport 12, 3553–3555. 10.1097/00001756-200111160-00036 11733710

[B6] Antal A. Nitsche M. A. Paulus W. (2006). Transcranial direct current stimulation and the visual cortex. Brain Res. Bull. 68, 459–463. 10.1016/j.brainresbull.2005.10.006 16459203

[B7] Batsikadze G. Moliadze V. Paulus W. Kuo M. F. Nitsche M. A. (2013). Partially non-linear stimulation intensity-dependent effects of direct current stimulation on motor cortex excitability in humans. J. Physiol. 591, 1987–2000. 10.1113/jphysiol.2012.249730 23339180 PMC3624864

[B8] Bikson M. (2015). Cellular mechanisms of tDCS: insights from animal models. Brain Stimul. 8:412 10.1016/j.brs.2015.01.313

[B9] Bikson M. Rahman A. Datta A. (2012). Computational models of transcranial direct current stimulation. Clin. EEG Neurosci. 43, 176–183. 10.1177/1550059412445138 22956646

[B10] Borckardt J. J. Bikson M. Frohman H. Reeves S. T. Datta A. Bansal V.. (2012). A pilot study of the tolerability and effects of high-definition transcranial direct current stimulation (HD-tDCS) on pain perception. J. Pain 13, 112–120. 10.1016/j.jpain.2011.07.001 22104190

[B11] Datta A. Bansal V. Diaz J. Patel J. Reato D. Bikson M. (2009). Gyri-precise head model of transcranial direct current stimulation: improved spatial focality using a ring electrode versus conventional rectangular pad. Brain Stimul. 2, 201.e1–207.e1. 10.1016/j.brs.2009.03.005 20648973 PMC2790295

[B12] Dmochowski J. P. Datta A. Bikson M. Su Y. Parra L. C. (2011). Optimized multi-electrode stimulation increases focality and intensity at target. J. Neural Eng. 8:046011. 10.1088/1741-2560/8/4/046011 21659696

[B13] Duijnhouwer J. Noest A. J. Lankheet M. J. Van Den Berg A. V. Van Wezel R. J. (2013). Speed and direction response profiles of neurons in macaque MT and MST show modest constraint line tuning. Front. Behav. Neurosci. 7:22. 10.3389/fnbeh.2013.00022 23576963 PMC3616296

[B14] Dumoulin S. O. Bittar R. G. Kabani N. J. Baker C. L. Le Goualher G. Pike G. B.. (2000). A new anatomical landmark for reliable identification of human area V5/MT: a quantitative analysis of sulcal patterning. Cereb. Cortex 10, 454–463. 10.1093/cercor/10.5.454 10847595

[B15] Engel S. A. Glover G. H. Wandell B. A. (1997). Retinotopic organization in human visual cortex and the spatial precision of functional MRI. Cereb. Cortex 7, 181–192. 10.1093/cercor/7.2.181 9087826

[B16] Faria P. Hallett M. Miranda P. C. (2011). A finite element analysis of the effect of electrode area and inter-electrode distance on the spatial distribution of the current density in tDCS. J. Neural Eng. 8:066017. 10.1088/1741-2560/8/6/066017 22086257 PMC3411515

[B17] Huberle E. Rupek P. Lappe M. Karnath H.-O. (2012). Perception of biological motion in visual agnosia. Front. Behav. Neurosci. 6:56. 10.3389/fnbeh.2012.00056 22973210 PMC3428581

[B18] Koch G. Fernandez Del Olmo M. Cheeran B. Schippling S. Caltagirone C. Driver J.. (2008). Functional interplay between posterior parietal and ipsilateral motor cortex revealed by twin-coil transcranial magnetic stimulation during reach planning toward contralateral space. J. Neurosci. 28, 5944–5953. 10.1523/jneurosci.0957-08.2008 18524898 PMC2648507

[B19] Maunsell J. H. Van Essen D. C. (1983). Functional properties of neurons in middle temporal visual area of the macaque monkey. I. Selectivity for stimulus direction, speed and orientation. J. Neurophysiol. 49, 1127–1147. 6864242 10.1152/jn.1983.49.5.1127

[B20] Minhas P. Bansal V. Patel J. Ho J. S. Diaz J. Datta A.. (2010). Electrodes for high-definition transcutaneous DC stimulation for applications in drug delivery and electrotherapy, including tDCS. J. Neurosci. Methods 190, 188–197. 10.1016/j.jneumeth.2010.05.007 20488204 PMC2920288

[B21] Minhas P. Datta A. Bikson M. (2011). Cutaneous perception during tDCS: role of electrode shape and sponge salinity. Clin. Neurophysiol. 122, 637–638. 10.1016/j.clinph.2010.09.023 21075048 PMC3053077

[B22] Miranda P. C. Faria P. Hallett M. (2009). What does the ratio of injected current to electrode area tell us about current density in the brain during tDCS? Clin. Neurophysiol. 120, 1183–1187. 10.1016/j.clinph.2009.03.023 19423386 PMC2758822

[B23] Nitsche M. A. Cohen L. G. Wassermann E. M. Priori A. Lang N. Antal A.. (2008). Transcranial direct current stimulation: state of the art 2008. Brain Stimul. 1, 206–223. 10.1016/j.brs.2008.06.004 20633386

[B24] Pirulli C. Fertonani A. Miniussi C. (2014). Is neural hyperpolarization by cathodal stimulation always detrimental at the behavioral level? Front. Behav. Neurosci. 8:226. 10.3389/fnbeh.2014.00226 25018709 PMC4073198

[B25] Richmond R. A. (2000). “Successful implementation of structured testing,” in IEEE International Test Conference (ITC) (Atlantic City, NJ), 344–348.

[B26] Stagg C. J. Lin R. L. Mezue M. Segerdahl A. Kong Y. Xie J.. (2013). Widespread modulation of cerebral perfusion induced during and after transcranial direct current stimulation applied to the left dorsolateral prefrontal cortex. J. Neurosci. 33, 11425–11431. 10.1523/jneurosci.3887-12.2013 23843514 PMC3724554

[B27] Tyler S. K. Tucker D. M. (1982). Anxiety and perceptual structure: individual differences in neuropsychological function. J. Abnorm. Psychol. 91, 210–220. 10.1037//0021-843x.91.3.210 7096791

[B28] Ungerleider L. G. Haxby J. V. (1994). ‘What’and ‘where’in the human brain. Curr. Opin. Neurobiol. 4, 157–165. 10.1016/0959-4388(94)90066-3 8038571

[B29] Van den Stock J. Tamietto M. Zhan M. Heinecke A. Hervais-Adelman A. Legrand L. B.. (2014). Neural correlates of body and face perception following bilateral destruction of the primary visual cortices. Front. Behav. Neurosci. 8:30. 10.3389/fnbeh.2014.00030 24592218 PMC3923138

[B30] Villamar M. F. Volz M. S. Bikson M. Datta A. Dasilva A. F. Fregni F. (2013). Technique and considerations in the use of 4x1 ring high-definition transcranial direct current stimulation (HD-tDCS). J. Vis. Exp. 77:e50309. 10.3791/50309 23893039 PMC3735368

[B31] Ward M. M. Stone S. C. Sandman C. A. (1978). Visual perception in women during the menstrual cycle. Physiol. Behav. 20, 239–243. 10.1016/0031-9384(78)90215-9 748933

[B32] Zimmermann P. Fimm B. (2002). “A test battery for attentional performance,” Applied Neuropsychology of Attention. Theory, Diagnosis and Rehabilitation, eds van Zomeren A. H. Leclercq M. Zimmermann P., (New York, NY: Psychology Press), 110–151.

[B33] Zito G. A. Müri R. Mosimann U. P. Nyffeler T. Nef T. (2014). A new method to measure higher visual functions in an immersive environment. Biomed. Eng. Online 13:104. 10.1186/1475-925x-13-104 25069675 PMC4118661

